# Temperature-Induced Annual Variation in Microbial Community Changes and Resulting Metabolome Shifts in a Controlled Fermentation System

**DOI:** 10.1128/mSystems.00555-20

**Published:** 2020-07-21

**Authors:** Shilei Wang, Wu Xiong, Yuqiao Wang, Yao Nie, Qun Wu, Yan Xu, Stefan Geisen

**Affiliations:** a State Key Laboratory of Food Science and Technology, Synergetic Innovation Center of Food Safety and Nutrition, School of Biotechnology, Jiangnan University, Wuxi, Jiangsu, China; b Department of Biology, Utrecht University, Utrecht, The Netherlands; c Laboratory of Nematology, Wageningen University, Wageningen, The Netherlands; d Department of Terrestrial Ecology, Netherlands Institute of Ecology NIOO-KNAW, Wageningen, The Netherlands; Purdue University

**Keywords:** fermentation system, microbiome, seasonal factors, temperature-induced

## Abstract

We are rapidly increasing our understanding on the spatial distribution of microbial communities. However, microbial functioning, as well as temporal differences and mechanisms causing microbial community shifts, remains comparably little explored. Here, using Chinese liquor fermentation as a model system containing a low microbial diversity, we studied temporal changes in microbial community structure and functioning. For that, we used high-throughput sequencing to analyze the composition of bacteria and fungi and analyzed the microbially derived metabolome throughout the fermentation process in all four seasons in both 2018 and 2019. We show that microbial communities and the metabolome changed throughout the fermentation process in each of the four seasons, with metabolome diversity increasing throughout the fermentation process. Across seasons, bacterial and fungal communities as well as the metabolome driven by 10 indicator microorganisms and six metabolites varied even more. Daily average temperature in the external surroundings was the primary determinant of the observed temporal microbial community and metabolome changes. Collectively, our work reveals critical insights into patterns and processes determining temporal changes of microbial community composition and functioning. We highlight the importance of linking taxonomic to functional changes in microbial ecology to enable predictions of human-relevant applications.

**IMPORTANCE** We used Chinese liquor fermentation as a model system to show that microbiome composition changes more dramatically across seasons than throughout the fermentation process within seasons. These changes translate to differences in the metabolome as the ultimate functional outcome of microbial activity, suggesting that temporal changes in microbiome composition are translating into functional changes. This result is striking as it suggests that microbial functioning, despite controlled conditions in the fermentors, fluctuates over season along with external temperature differences, which threatens a reproducible food taste. As such, we believe that our study provides a stepping-stone into novel taxonomy-functional studies that promote future work in other systems and that also is relevant in applied settings to better control surrounding conditions in food production.

## Abstract (precis)

We used Chinese liquor fermentation as a model system to show that microbiome composition changes more dramatically across seasons than throughout the fermentation process within seasons. These changes translate to differences in the metabolome as the ultimate functional outcome of microbial activity, suggesting that temporal changes in microbiome composition are translating into functional changes. This result is striking as it suggests that microbial functioning, despite controlled conditions in the fermentors, fluctuates over season along with external temperature differences, which threatens a reproducible food taste. As such, we believe that our study provides a stepping-stone into novel taxonomy-functional studies that promote future work in other systems and that also is relevant in applied settings to better control surrounding conditions in food production.

## INTRODUCTION

Microorganisms are the most abundant biota and compose the second-largest biomass source on the planet (1). At a functional level, microorganisms determine human and environmental health (2, 3) and agricultural production (4). Therefore, large-scale studies aiming at identifying microbial community compositions in different environments have been carried out (5, 6). These studies have revealed that multiple environmental factors drive microbial community changes at large spatial scales, including pH (7), temperature (8), moisture (9), and salinity (10). For example, pH and temperature as the main environmental factors drive bacterial community composition in soils (7, 11), while latitude and soil carbon determine fungal community compositions (12). In other systems, such as in strongly human-controlled fermentation systems, temperature is the key driver of microbial communities with bacteria being more affected than fungi (13, 14).

Most studies, such as the ones mentioned above, have focused on spatial scale variation and sampled at a given geographic point a single temporal sample. Yet, the high growth rate and adaptation potential of microorganisms induce rapid temporal changes (15–17). Several climatic factors that are known to influence microbial community composition fluctuate over seasons, such as temperature (18) and precipitation (19), which therefore likely determine community profiles in a similar way as spatial patterns. For example, in agricultural soils, abiotic properties and therefore bacterial and fungal communities change throughout seasons (20). Both extent and main determinants of temporal changes remain, however, mostly unknown at microbial community levels. Furthermore, it is commonly unknown if, and to what extent, environmental factors influence microbiomes in human-controlled systems at larger temporal scales.

Changes in microbial community profiles can affect microbial functioning (21), as the production of secondary metabolites is commonly linked to microbial community profiles (22). Even individual microorganisms adapt their functioning in response to changes in their surroundings through differential gene expression, resulting in a change of their expressed metabolites (23, 24). Many of these microbe-derived metabolites are used in a variety of ways for human purposes (25), such as to produce antibiotics (26), ethanol (27), and organic acids (28). Yet, we are often limited in our understanding, especially in complicated community settings, of how changes in microbial composition translate to functional changes that are visible in the metabolome profile. Microbe-derived functions are, however, not always linked to changes in entire community profiles but are determined by individual keystone species (29), such as Plasmodium falciparum as the causal agent of malaria (30) and specific fungal pathogens that can diminish crop yields (31). Also, in human-used fermentation systems, individual keystone microbes determine functions and applications, such as *Saccharomyces* for the production of beer (32), wine (33), and bread (34) and *Lactobacillus* spp. in milk fermentation (35).

To study temporal variations and their determinants within and across seasons in microbial communities and potentially underlying microbial functioning, we chose Chinese liquor fermentation as a model system. The Chinese liquor fermentation system is specified by a rather simple community of environmentally originating microorganisms which survive the harsh conditions and drive spontaneous fermentation (36–39). Due to their functional importance, these comparably few microorganisms driving fermentation are well known (40), such as the human gut microorganisms (15). Microbial communities in Chinese liquor fermentation have recently been shown to differ between seasons (41), but it remains unknown if the community changes would induce the changes in microbial functioning through altering the metabolome (42). We collected samples in a liquor distillery throughout the fermentation process (5 sampling points up to 25 days after initiating fermentation) in all four seasons, tracked and recorded six major seasonally fluctuating climate factors (atmospheric pressure, daily average temperature, relative humidity, precipitation, wind speed, and sunshine duration) (43), determined bacterial and fungal communities using amplicon sequencing, and identified the metabolome in all samples. This information allowed us to investigate if and how microbial communities and their metabolome change within and across seasons, as well as to determine the key factors underlying these changes. We hypothesized that (hypothesis 1) microbial communities differ within and between seasons (and we expected that microbial communities change more strongly throughout the fermentation process than between seasons); (hypothesis 2) microbial changes are more pronounced in bacterial than in fungal communities; (hypothesis 3) functions change in the metabolome throughout the fermentation process within individual seasons while metabolome profiles remain the same across seasons to ensure a reproducible taste; (hypothesis 4) microbial community changes are driven by external environmental conditions, particularly fluctuations in temperature; and (hypothesis 5) observed metabolic changes are driven by specific indicator microorganisms rather than the whole community and therefore can be predicted by changes of the individual indicator taxa.

## RESULTS

### Overall microbial composition at different seasonal scales.

In our targeted investigation of 213 samples during liquor fermentation across four seasons from 2018 to 2019 (see Table S1 in the supplemental material), we found that bacterial operational taxonomic unit (OTU) number and diversity were significantly (*P < *0.05) lower in summer than in the other three seasons, as shown by the indices of Chao1 (Fig. S1a and Table S2) and Shannon (Fig. S1b and Table S2). In contrast, fungal Chao1 (Fig. S1c and Table S3) and Shannon diversity (Fig. S1d and Table S3) in winter were significantly (*P < *0.05) lower than in the other three seasons.

10.1128/mSystems.00555-20.1 FIG S1 Diversity difference. (a) Chao1 index of bacteria in seasonal scales; (b) Shannon index of bacteria in seasonal scales; (c) Chao1 index of fungi in seasonal scales; (d) Shannon index of fungi in seasonal scales; (e) Chao1 index of bacteria in fermentation time; (f) Shannon index of bacteria in fermentation time; (g) Chao1 index of fungi in fermentation time; (h) Shannon index of fungi in fermentation time. Download FIG S1, PDF file, 0.1 MB. Copyright © 2020 Wang et al. 2020 Wang et al. This content is distributed under the terms of the Creative Commons Attribution 4.0 International license.

10.1128/mSystems.00555-20.4 TABLE S1 Sample collection information and daily variation of seasonal factors. Download Table S1, XLSX file, 0.02 MB. Copyright © 2020 Wang et al. 2020 Wang et al. This content is distributed under the terms of the Creative Commons Attribution 4.0 International license.

10.1128/mSystems.00555-20.5 TABLE S2 Bacterial alpha diversity. Download Table S2, XLSX file, 0.02 MB. Copyright © 2020 Wang et al. 2020 Wang et al. This content is distributed under the terms of the Creative Commons Attribution 4.0 International license.

10.1128/mSystems.00555-20.6 TABLE S3 Fungal alpha diversity. Download Table S3, XLSX file, 0.02 MB. Copyright © 2020 Wang et al. 2020 Wang et al. This content is distributed under the terms of the Creative Commons Attribution 4.0 International license.

**FIG 1:**
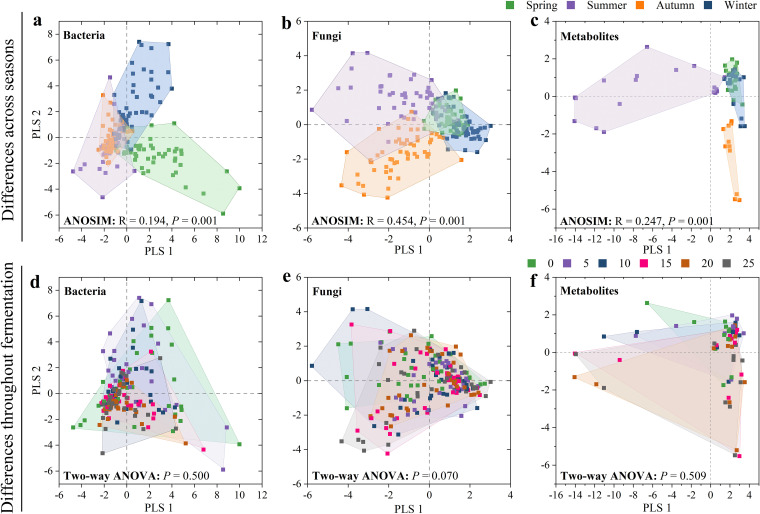
Partial least square-discriminant analysis (PLS-DA) for bacteria, fungi, and metabolites. (a to c) Effect of season on bacterial communities (a), fungal communities (b), and metabolites (c) as analyzed using ANOSIM. (d to f) Effect of fermentation time on bacterial communities (d), fungal communities (e), and metabolites (f) as analyzed using two-way ANOVA.

Using partial least square-discriminant analysis (PLS-DA), we found that bacteria (analysis of similarity [ANOSIM]: *R* = 0.194, *P = *0.001) (Fig. 1a) and fungi (ANOSIM: *R* = 0.454, *P = *0.001) (Fig. 1b) showed significant differences at the different seasons. The diversity of both bacteria and fungi decreased throughout fermentation from 0 to 25 days (Fig. 1d and e), but these changes were not statistically significant. However, there were significant differences (*P < *0.05) in the diversity of bacteria and fungi under fermentation conditions on the same day in different seasons (Fig. S1e to h). For example, during the fermentation, the Chao1 index of bacteria in summer was significantly (*P < *0.05) lower than in other seasons (Fig. S1e).

**FIG 2:**
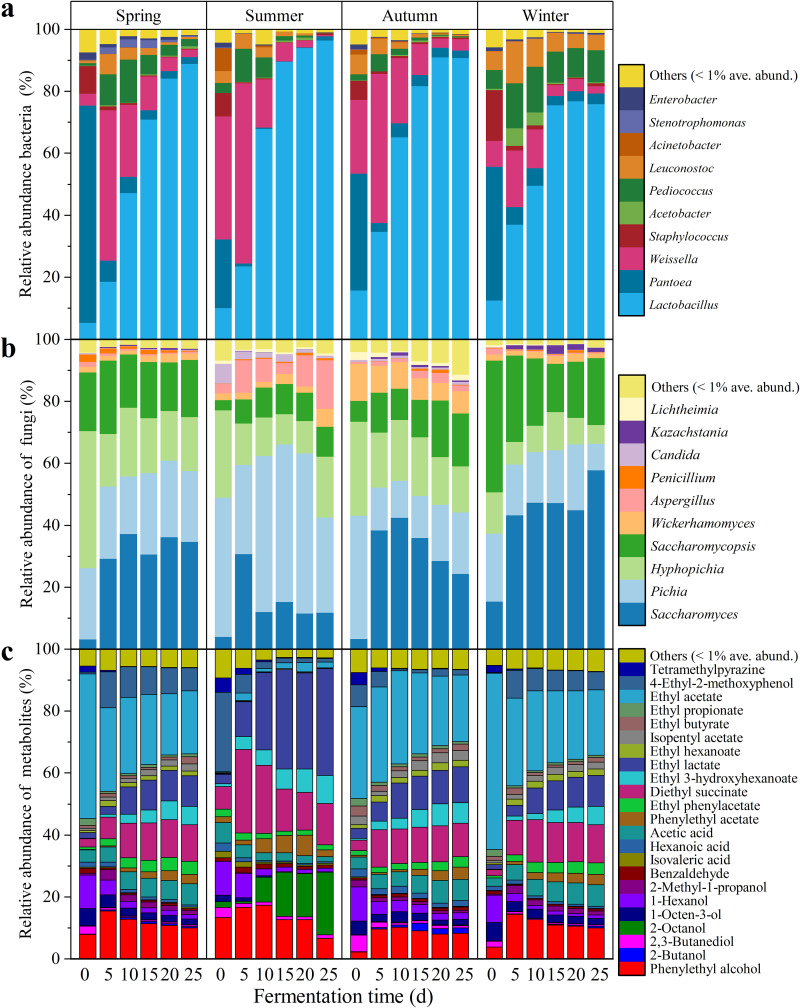
Relative abundance of bacteria (a), fungi (b), and metabolites (c) at different seasons (*n* = 9). Only those genera and metabolites that had an average abundance greater than 1% in at least one season are indicated. Genera with less than 1% abundance are combined and shown in the “others” category. Four successive seasons (spring, summer, autumn, and winter) compose a complete seasonal cycle. The unit of fermentation time is “d” for days.

Across all communities sampled, 20 dominant genera (average abundance ≥1%) were shared across all seasons, while their relative abundances varied between seasons (*P < *0.05) (Fig. 2a and b).

### Overall function composition at different seasonal scales.

We further collected the metabolomes of microbial communities across seasons and detected a total of 49 metabolites with 23 metabolites being abundant (relative abundance over 1%) (Fig. 2c). The metabolome (ANOSIM: *R* = 0.247, *P = *0.001) (Fig. 1c) significantly differed between seasons. For example, ethyl lactate and 2-octanol were significantly higher (*P < *0.05) in summer than in other seasons. The diversity of metabolites increased over time, suggesting that metabolites accumulated (Fig. 1f).

### Indicator microorganisms and their metabolites across seasons.

**FIG 3:**
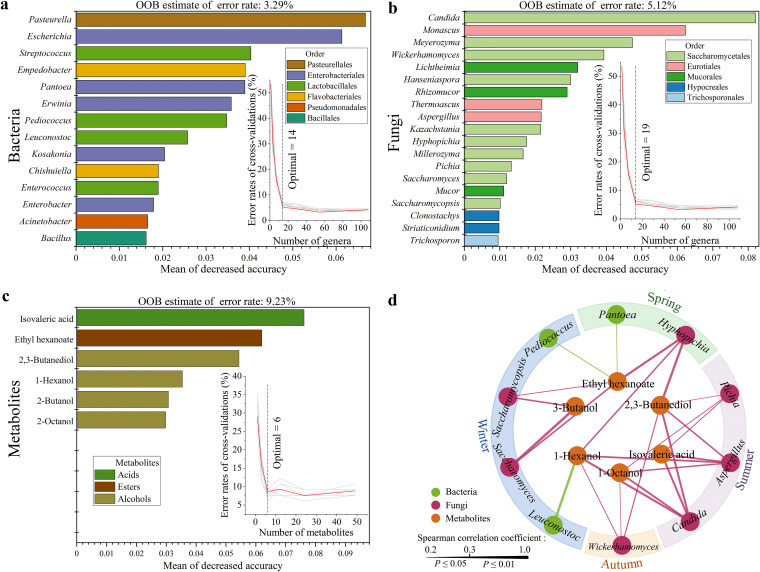
Analysis of seasonal indicator microorganisms and metabolites by random forest modeling. (*n* = 9). (a) Bacteria. (b) Fungi. (c) Metabolites. OOB (out of the bag) is an estimate of the error rate. The mean for decreased accuracy represents the decreasing degree of the accuracy of the random forest prediction. (d) Correlation network between seasonal indicator microorganisms and metabolites during the fermentation process. Line thickness is proportional to the value of Spearman’s correlation. Line color represents different seasons (spring, green; summer, purple; autumn, orange; winter, blue). Outer circle nodes represent microorganisms; inner circle nodes represent metabolites.

The random forest machine learning approach revealed that the accuracies of predicting indicator bacteria, fungi, and metabolites were 96.71%, 94.88%, and 90.77%, respectively (Fig. 3a to c). For microbial taxa, the cross-validation error curve stabilized when the 14 and 19 most relevant bacteria and fungi, respectively, were included (Fig. 3a and b), in which the bacterial genera *Pantoea*, *Pediococcus*, and *Leuconostoc* (Fig. S2a) and the fungal genera *Aspergillus*, *Candida*, *Hyphopichia*, *Pichia*, *Saccharomyces*, *Saccharomycopsis*, and *Wickerhamomyces* (Fig. S2b) were the dominant genera. Using only these taxa, our model accurately predicted bacteria (accuracy ≥96.08%) and fungi (accuracy ≥92.59%) in the confusion matrix (Fig. S2c and d). Thus, we defined these 10 genera (3 being bacteria and 7 being fungi) as seasonal indicator genera.

10.1128/mSystems.00555-20.2 FIG S2 Identification of dominant seasonal indicator microorganisms and confusion matrix inspection. (a) Dominant seasonal indicator bacteria. (b) Dominant seasonal indicator fungi. (c) Confusion matrix inspection of bacteria. (d) Confusion matrix inspection of fungi. (e) Confusion matrix inspection of metabolites. Download FIG S2, PDF file, 0.1 MB. Copyright © 2020 Wang et al. 2020 Wang et al. This content is distributed under the terms of the Creative Commons Attribution 4.0 International license.

For microbial metabolites, the cross-validation error curve stabilized when the 6 most abundant metabolites were used (Fig. 3c). Thus, we defined these 6 metabolites (isovaleric acid, ethyl hexanoate, 2,3-butanediol, 1-hexanol, 2-butanol, and 2-octanol) as seasonal indicator metabolites. Our model accurately (accuracy ≥83.33%) predicted metabolites in the confusion matrix (Fig. S2e). All 10 seasonal indicator genera significantly (*P < *0.05) correlated with the six seasonal indicator metabolites (Fig. 3d and Table S4).

10.1128/mSystems.00555-20.7 TABLE S4 Correlation analysis of microorganisms and metabolites. Significance levels are denoted with *** ≤0.001 < ** ≤0.01 < * ≤0.05. Download Table S4, XLSX file, 0.01 MB. Copyright © 2020 Wang et al. 2020 Wang et al. This content is distributed under the terms of the Creative Commons Attribution 4.0 International license.

### Key seasonal factor determining microbial and metabolite indicators.

**FIG 4:**
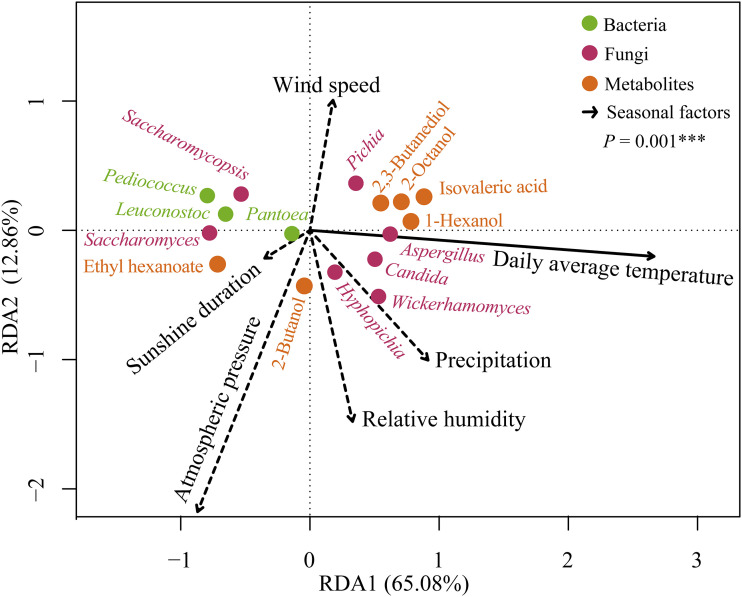
RDA of seasonal factors, indicator microorganisms, and metabolites (*n* = 9). Percentages on the axes represent the eigenvalues of principal components. Green dots represent bacteria, red dots represent fungi, and yellow dots represent metabolites. Black arrows point to different seasonal factors. The solid line indicates that there is a significant (*P ≤ *0.01) correlation between seasonal factor and indicators, while dotted lines indicate that there is no significant (*P* > 0.05) correlation between seasonal factors and indicators. Significance levels are denoted with 0 < *** ≤0.001 < ** ≤0.01 < * ≤0.05.

Redundancy analysis (RDA) was used to identify the key seasonal factor that determines indicator microorganisms and metabolites. We showed significant correlations (*P = *0.001) between 6 seasonal factors and 16 seasonal indicators (10 microorganisms and 6 metabolites) (Fig. 4). Daily average temperature was the key seasonal factor determining bacteria (*P < *0.001, relative importance [RI] = 90.07%), fungi (*P = *0.003, RI = 51.09%), and metabolites (*P = *0.002, RI = 51.73%) (Table S5).

10.1128/mSystems.00555-20.8 TABLE S5 Relative importance of indicators. Significance levels are denoted with *** ≤0.001 < ** ≤0.01 < * ≤0.05. Download Table S5, XLSX file, 0.01 MB. Copyright © 2020 Wang et al. 2020 Wang et al. This content is distributed under the terms of the Creative Commons Attribution 4.0 International license.

### Predicting seasonal indicators based on daily average temperature.

**FIG 5:**
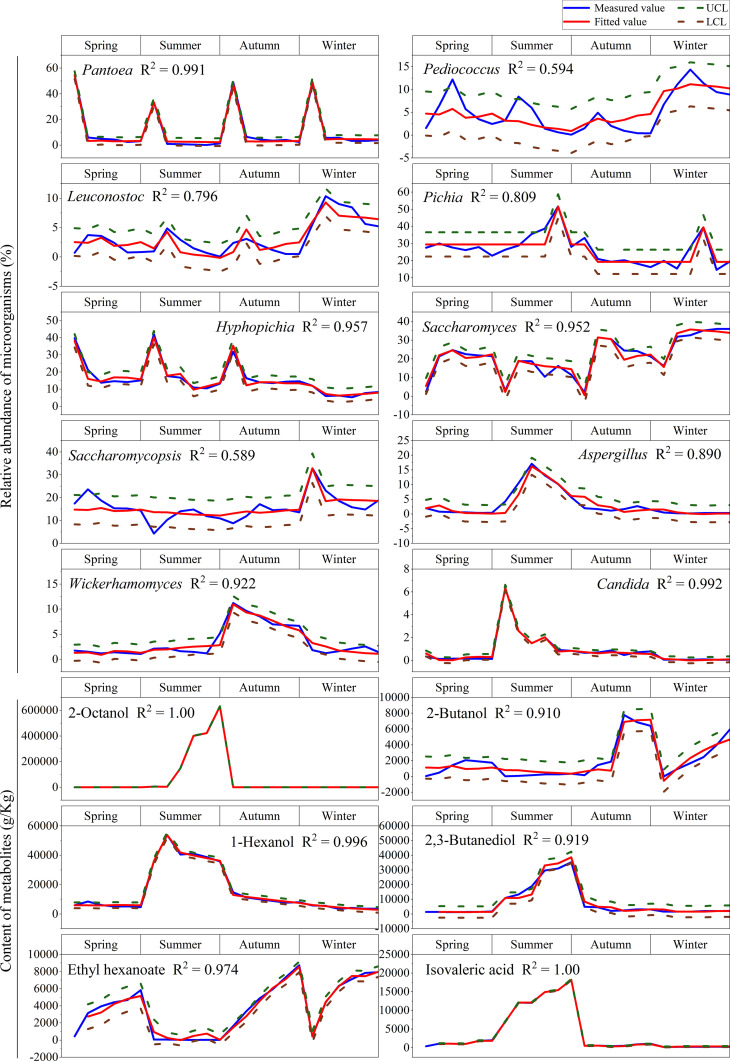
Prediction model of time series (*n* = 9). The blue line is the measured values, and the red line is the fitted values. UCL, upper confidence limit (green). LCL, lower confidence limit (brown).

We further confirmed daily average temperature as the key seasonal factor determining the microbial composition and functioning by an autoregressive moving average (ARMA) model (Fig. 5 and Table S6). The ARMA model revealed that *R*^2^ values of all 16 curves were above 0.5 (accuracy = 100%) and for 11 curves were above 0.9 (accuracy = 68.75%).

10.1128/mSystems.00555-20.9 TABLE S6 Autoregressive moving average (ARMA) model statistics. Download Table S6, XLSX file, 0.01 MB. Copyright © 2020 Wang et al. 2020 Wang et al. This content is distributed under the terms of the Creative Commons Attribution 4.0 International license.

Each seasonal indicator genus and metabolite corresponded to four seasons, so 16 indicators corresponded to 64 seasonal points. According to the model driven by the daily average temperature (Fig. 5), 61 of 64 seasonal points could be predicted with an accuracy rate of 95.31%. Autocorrelation (ACF) and partial autocorrelation (PACF) showed that almost all defined indicators were within the limited range of residuals (residuals <0.5) in the prediction model (Fig. S3).

10.1128/mSystems.00555-20.3 FIG S3 Residual error of prediction model. ACF, autocorrelation; PACF, partial autocorrelation curve. Download FIG S3, PDF file, 0.1 MB. Copyright © 2020 Wang et al. 2020 Wang et al. This content is distributed under the terms of the Creative Commons Attribution 4.0 International license.

## DISCUSSION

Microbial community composition differed within and between seasons, but in contrast to hypothesis 1, microbial communities changed more strongly between than within seasons. Numerous studies have shown that environmental factors that change across seasons can cause differences in microbial community composition (44, 45). We found that these microbial changes were more pronounced in bacterial than in fungal communities, consistent with hypothesis 2 and previous studies (13, 14). The reduction of bacterial diversity in summer compared with other seasons supported this finding.

Differences in microbiome composition across temporal scales translated into differences in the metabolome—such as microbial functioning—as proposed before (46). This finding, however, challenges hypothesis 3 and suggests that functional differences might even result in changes in human applications, here taste. Indeed, microbe-derived functions, such as metabolites in this study, directly translate to human applications (47). The link between taxonomic and functional changes has previously been observed also in other systems such as in soils (48, 49). As such, it is of great significance to study community differences and link them to functioning to evaluate the functional consequences of observed community changes in any studied system.

More and more research focuses on potential microbial function based on the presence of functional genes such as in metagenomic or metatranscriptomic approaches in different environmental settings as well as human hosts (22, 50). However, this research provides only limited information on microbial functioning as gene presence (DNA-based analyses) or expression (RNA-based analyses) often does not directly translate to successful protein translation or even metabolite production and, as such, microbial functioning (51). Indeed, only the metabolome represents a synonym for microbial functioning (50). We urge future studies to adapt an approach as done here to link microbial communities to function to better understand mechanisms of microbial community functioning.

The observed microbial and functional changes were driven by changes in external environmental conditions, particularly temperature, confirming hypothesis 4. Indeed, the temperature is an essential factor that can change microbial population dynamics (52–54). Changes in the taxonomic composition of microbial communities reflected by changes in abiotic conditions are in line with previous studies, focusing on spatial-scale microbial community patterns (46, 55). Thus, abiotic habitat fluctuations also determine temporal changes in microbial communities (56). This suggests that the temperature-controlled fermentation system is still massively influenced by fluctuations in the surrounding environment that determine microbial assembly and function even in highly controlled fermentation systems.

Finally, we found that observed metabolic changes are driven by specific indicator microorganisms and therefore can be predicted by changes of individual indicator taxa, which is in line with hypothesis 5. Similar concepts are also mentioned in different studies that claim the importance of keystone species (57) or core microbiota (58) as underlying drivers of functional changes. Thus, it is helpful to simplify complex communities into individual taxa to determine and focus subsequent analyses on functionally important keystone species (59, 60). In comparison to commonly performed whole-community analyses (8, 48, 61), the method based on indicator microorganisms and metabolites can accurately reflect changes in environmental conditions. Our indicator taxon approach to determine the key factors driving community and functional changes not only could reveal differences much more strongly but even allowed accurate modeling of temporal changes in taxonomically important microorganisms and functional metabolites. As such, we believe that our approach might replace whole-community analyses to forecast both taxonomic and functional changes as individual taxonomic groups and functions (metabolites) determine system functioning.

These results indicate that microbial functioning, despite controlled conditions in the fermentors, fluctuates over season along with external temperature differences which influence consistent food taste. Based on our predictive model, it is possible to compensate for the adverse effects of seasonal factors in the future under different temperature conditions by manual intervention in indicator microorganisms. As such, our study provides a stepping-stone into novel taxonomy-function studies. This was possible only using Chinese liquor fermentation as a model system as all taxa present in these simple settings are functionally well characterized. Based on our approach, studies can now be extended to involving more complex microbial communities.

### Conclusion.

Together, this study increases our understanding of links between microbial community profiles and functions at various temporal scales, such as within and across seasons. This information can be used to improve food quality by inducing targeted microbiome changes through a change in temperature of not only the fermentation containers but also the surroundings of the fermentors. The knowledge obtained in this model system should also stimulate research in other environments to enable tailor-made manipulation of microbial communities and their functioning.

## MATERIALS AND METHODS

### Sample collection.

We collected samples spanning the entire fermentation process (days 0, 5, 10, 15, 20, and 25) in all four seasons in 2018 and 2019 in a liquor distillery (Qinghai Huzhu Highland Barley Distillery Co. Ltd., Qinghai, China). For solid-state fermentation, steamed grains were mixed with a starter (fermentation products containing a variety of microorganisms) at a ratio of 9:1 (wt/wt) and fermented in a sealed tank for 25 days (62). We collected a total of 213 samples (100 g) from 36 jars in the center of the layer (0.5 m deep) at different fermentation times (days 0, 5, 10, 15, 20, and 25). All samples were stored at −20°C for further DNA extraction and physicochemical parameter determination.

### DNA extraction, qualification, and sequencing analysis.

Five-gram subsamples were used to extract total genomic DNA using the E.Z.N.A. soil DNA kit (Omega Bio-Tek, Norcross, GA) according to the manufacturer’s instruction. The V3-V4 region of the 16S rRNA gene was amplified with the universal primers 338F/806R to target bacteria (63). For fungi, the ITS2 region was amplified with the primers ITS2/ITS3 (64). A unique 8-nucleotide barcode sequence was added to the primers to differentiate between samples. PCRs were performed in 25-μl volume tubes, containing 2.5 μl of 10× Pyrobest buffer, 2 μl of 2.5 mM deoxynucleoside triphosphates (dNTPs), 1 μl of each primer (10 μM), 0.4 U of Pyrobest DNA polymerase (TaKaRa, TaKaRa Holdings Inc., Nojihigashi, Kusatsu, Shiga, Japan), 15 ng of template DNA, and double-distilled water (ddH_2_O) up to 25 μl. Amplification was performed as described before (65, 66). Amplicons were pooled in equimolar quantities and were subjected to high-throughput sequencing using MiSeq sequencing for 2- by 300-bp paired-end sequencing (Illumina, San Diego, CA).

### Sequence processing.

All raw sequences were processed via QIIME (V. 1.8) (67). Briefly, high-quality sequences were obtained by removing sequences with ambiguous bases <2, homopolymers <10, primer mismatches, average quality scores <20, and lengths (excluding the primer or barcode region) <50 bp. Chimeras were removed using USEARCH (V. 10) (68). Using Uclust (V. 1.2.22), we clustered the trimmed sequences into operational taxonomic units (OTUs) with 97% sequence similarity (69). Databases EzBioCloud (www.ezbiocloud.net, a public database of bacterial sequences) and Central Bureau of Fungal Cultures (CBS-KNAW, www.wi.knaw.nl, a public database of fungal sequences) were used for sequence alignment of bacterial 16S rRNA genes and fungal ITS2 regions, respectively (27).

### Analysis of metabolites.

Five-gram subsamples were added to 10 ml ddH_2_O, placed in an ultrasonic cleaner (AS30600B; Autoscience, Tianjin, China) for 30 min, and centrifuged at 8,000 × *g* for 10 min. After filtering using an 0.2-μm-pore-size filter, the filtrate was used to analyze metabolite concentrations (27). Metabolites were detected using gas chromatography-mass spectrometry (Agilent 6890N GC system and Agilent 5975 mass selective detector; Agilent, Santa Clara, CA) with condition details based on a previous study (65).

### Climate data.

The daily weather data were collected from the Qinghai Huzhu ground meteorological automatic station (ZXCAWS630; Zxweather, Beijing, China). Daily measurements were extracted for atmospheric pressure, daily average temperature, relative humidity, precipitation, wind speed, and sunshine duration in 2018 and 2019.

### Statistical analysis.

Statistical analyses and diversity analysis were conducted with XLSTAT (V. 19.02.42992). We calculated Shannon index and Chao1 estimator using QIIME (V. 1.8) (70, 71). Partial least square-discriminant analysis (PLS-DA) was used to calculate differences of bacterial and fungal genera and metabolites between seasons and throughout fermentation in R (V. 3.5.0) (72). In order to determine differences within and between seasons, two-way analysis of variance (ANOVA) and ANOSIM were used to test significant differences between sample groups based on Bray-Curtis distance matrices (43). We identified seasonal indicator genera and metabolites by the Random Forest package (V. 4.6-14) in R (73). Cross-validation was performed by the “rfcv” function for selecting appropriate features. The “varImpPlot” function was used to show the importance of features in the classification. The importance of features and the cross-validation curve were visualized using ggplot2 (V. 2.2.1) in R. Spearman correlation coefficients (ρ) were calculated with SPSS Statistics 25. Creation of visualizations of metabolites and genus interactions and cooccurring analysis were performed with Gephi (V. 0.9.1) (74). Redundancy analysis (RDA) was used to identify the relationship between environmental factors and microbes, and analyses were performed in Vegan (V. 2.4-3) (75). The relative importance of seasonal factors was determined in “relaimpo” (V.2.2-3) (76). We chose the autoregressive moving average (ARMA) model to verify the accuracy of the seasonal factors. The ARMA model is a linear model associated with stationary time series with the minimum number of parameters (77). We used daily average temperature as a parameter to model and predicted seasonal marker genera and metabolites in SPSS Statistics 25 (78).

### Data availability.

Bacterial and fungal raw sequence data were deposited in the DNA Data Bank of Japan (DDBJ) database under the accession numbers DRA009350 and DRA009351.
