# Supplementary figures and images for: Temperature-Induced Annual Variation in Microbial Community Changes and Resulting Metabolome Shifts in a Controlled Fermentation System
Source: mSystems. 2020 Jul 21;5(4):e00555-20. doi: 10.1128/mSystems.00555-20 (PMC7566281; doi:10.1128/mSystems.00555-20)

Differences across seasons

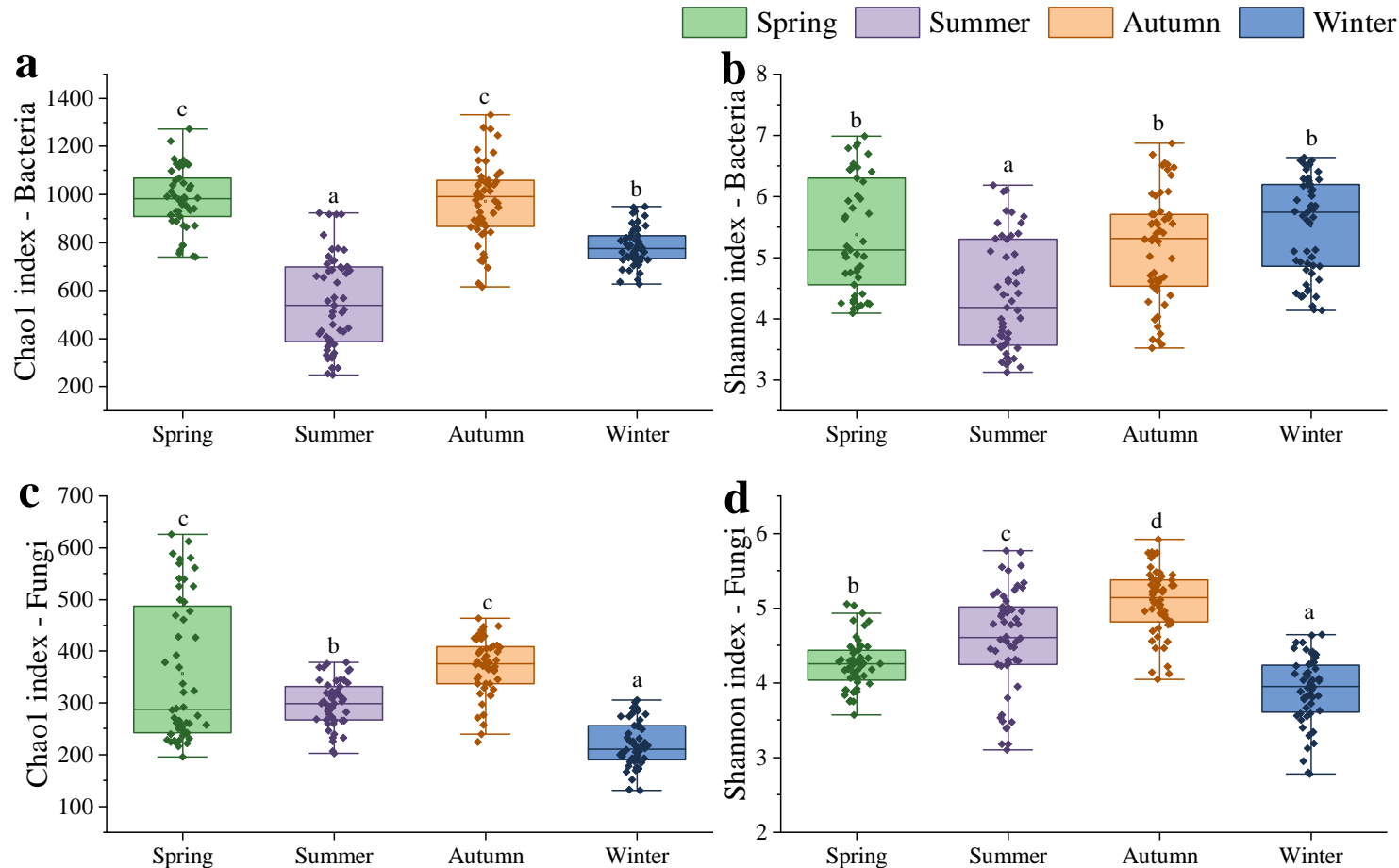

Difference through fermentation

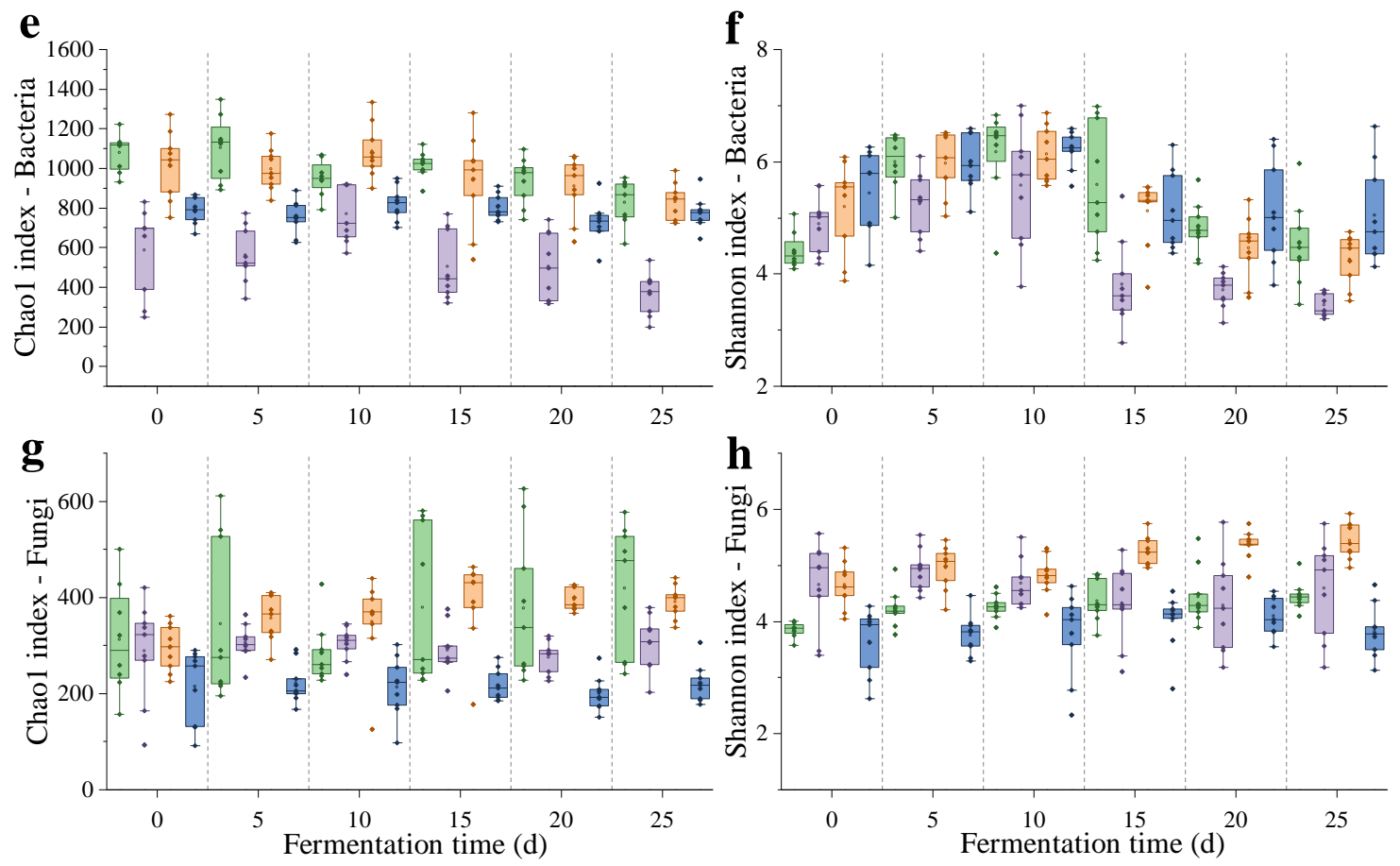

Supplement: FIG S1 [file mSystems.00555-20-sf001.pdf]

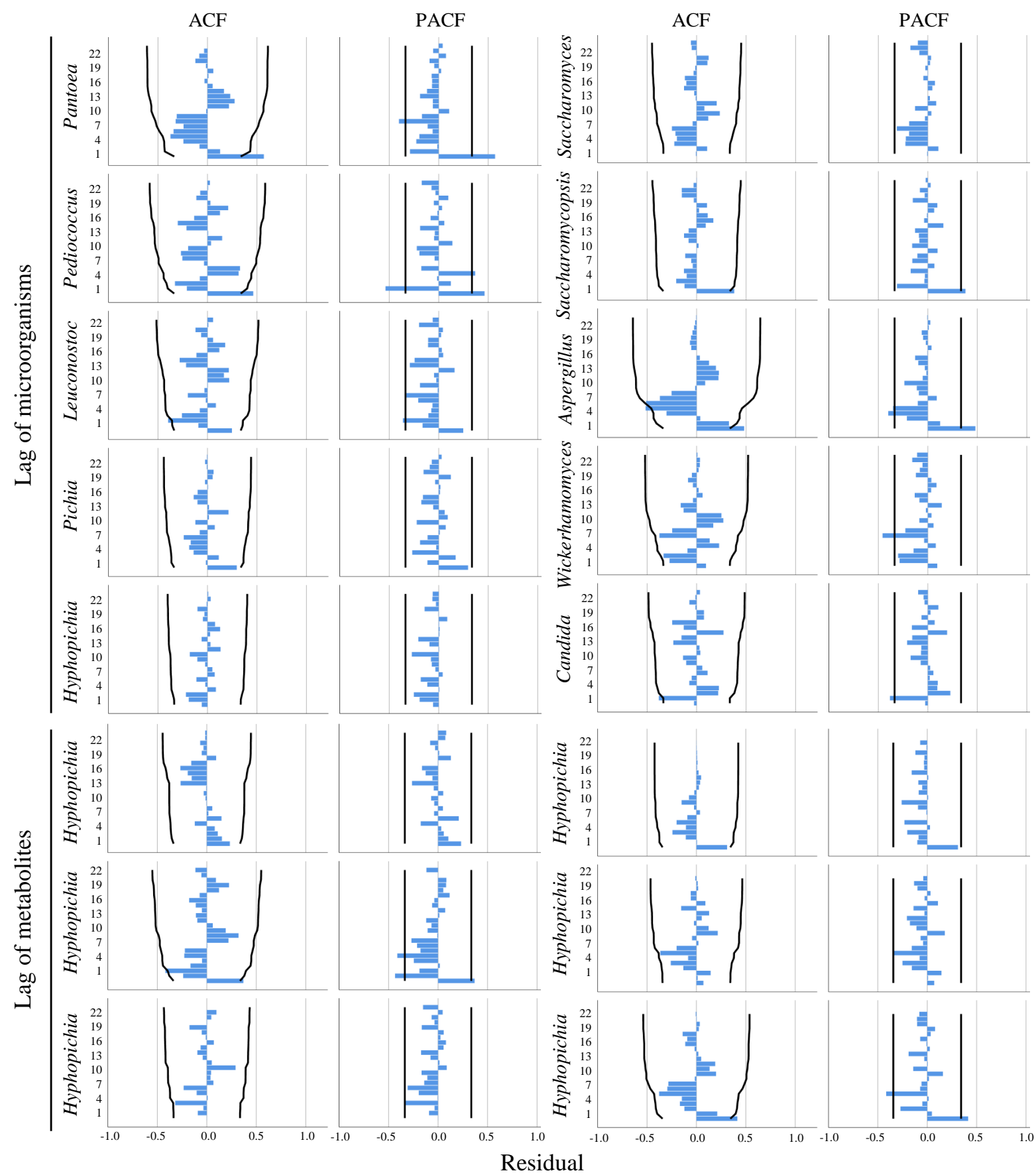

Supplement: FIG S3 [file mSystems.00555-20-sf003.pdf]
