# Supplementary material for: Temperature-Induced Annual Variation in Microbial Community Changes and Resulting Metabolome Shifts in a Controlled Fermentation System
Source: mSystems. 2020 Jul 21;5(4):e00555-20. doi: 10.1128/mSystems.00555-20 (PMC7566281; doi:10.1128/mSystems.00555-20)
Supplement: FIG S2 [file mSystems.00555-20-sf002.pdf]

a

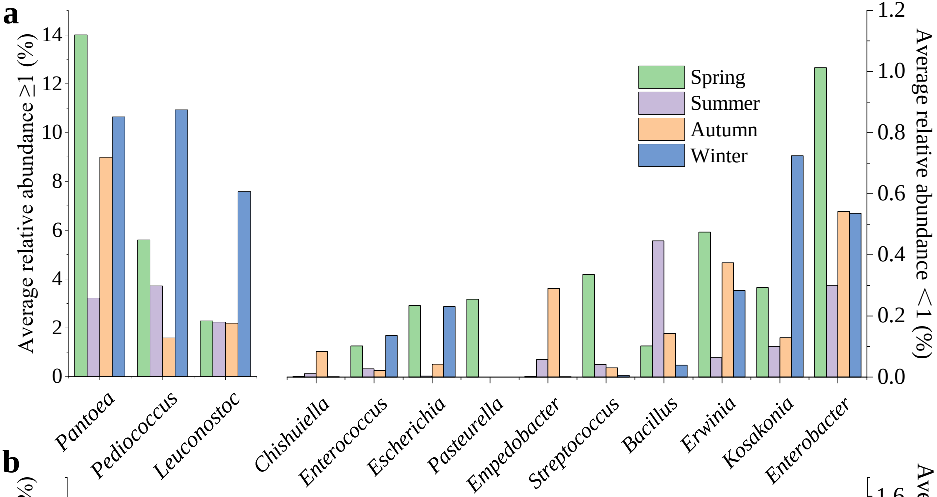

b

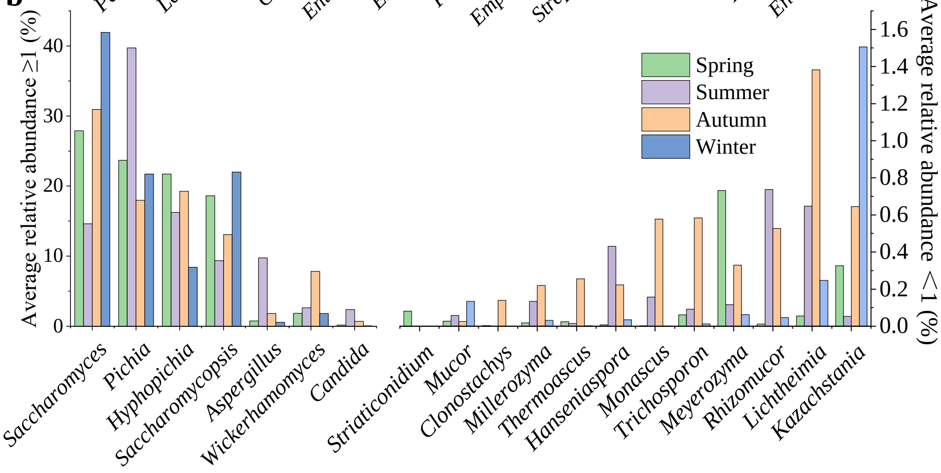

c Confusion matrix:

Season	Spring	Summer	Autumn	Winter	Class. error (%)
Spring	49	1	0	1	3.92
Summer	0	52	2	0	3.70
Autumn	0	2	52	0	3.70
Winter	0	1	0	53	1.85

d Confusion matrix:

Season	Spring	Summer	Autumn	Winter	Class. error (%)
Spring	50	0	0	3	5.66
Summer	0	52	2	0	3.70
Autumn	0	3	50	1	7.41
Winter	1	0	0	53	1.85

e Confusion matrix:

Season	Spring	Summer	Autumn	Winter	Class. error (%)
Spring	11	0	0	1	8.33
Summer	0	15	0	3	16.67
Autumn	0	0	18	0	0.00
Winter	0	2	0	15	11.76
